# Inherent humic substance promotes microbial denitrification of landfill leachate via shifting bacterial community, improving enzyme activity and up-regulating gene

**DOI:** 10.1038/s41598-017-12565-3

**Published:** 2017-09-22

**Authors:** Shanshan Dong, Mu Li, Yinguang Chen

**Affiliations:** State Key Laboratory of Pollution Control and Resource Reuse, College of Environmental Science and Engineering, Tongji University, 1239 Siping Road, Shanghai, 200092 China

## Abstract

Microbial denitrification is the main pathway for nitrogen removal of landfill leachate. Although humic substances (HSs) have been reported in landfill leachate, the effects of HS on denitrification process of activated sludge for leachate treatment are still unknown. In this study, we adopted SAHA as the model HS to study the effects of HS on the denitrification of landfill leachate. After long-term operation at 10 mg/L of Shanghai Aladdin Humic Acid (SAHA), the final nitrate concentration and nitrite accumulation were much lower than the control (5.2 versus 96.2 mg/L; 0.5 versus 34.7 mg/L), and the final N_2_O emission was 13.1% of the control. The mechanistic study unveiled that SAHA substantially changed the activated sludge community structure and resulted in the dominance of *Thauera* after long-term exposure to SAHA. *Thauera* could be able to utilize HSs as electron shuttle to improve denitrificattion performance, especially for nitrite reduction. Moreover, SAHA significantly upregulated the gene expressions and catalytic activities of the key enzymes related to denitrification, the reducing power (NADH) generation, and the electron transport system activity, which accelerated nitrogen oxide reduction. The positive effects of HS on denitrification performance were confirmed by the addition of SAHA into real leachate.

## Introduction

Large amounts of leachates were generated from sanitary landfill treatment of municipal solid waste (MSW)^1^. In general, leachates are composed of biodegradable organics, recalcitrant organics (where humic substances constitute a key group), ammonium nitrogen, heavy metals and some inorganic salts. Leachates need to be carefully treated before discharge because the high concentrations of organic matters and ammonium (always several hundreds to thousands mg/L) can produce the large danger and harm to the environment^2–4^. The biological processes are suitable to treat leachate for their reliability, simplicity and economy^5^. During biological processes, the nitrogen removal of landfill leachate is achieved through nitrification and denitrification, which is carried out by ammonium-oxidizing bacteria and nitrite-oxidizing bacteria, and denitrifying bacteria, respectively^6^. In short, ammonium is initially oxidized to nitrite and then to nitrate, and nitrate is later anaerobically reduced to nitrite, nitric oxide, nitrous oxide, and the final nitrogen gas, which means the real nitrogen removal from leachate.

Humic substances (HSs) are important components of the natural organic matter in the environment, including landfill leachate, soils and sediments, etc^7^. HS is a kind of supramolecular-weight aromatic polymer which made up of cross-linked alkyl or aromatic units, with the major functional groups: carboxylic acid and quinone moieties, etc^8,9^. It is thought that redox-reactive quinone moieties in HS can act as electron shuttles or donors to reduce the inorganic and organic compounds by chemical and microbial reaction^10,11^. Derek R. Lovely *et al*.^12^ found the dissimilatory Fe(III)-reducing bacterium *Geobacter metallireducens* could obtain electrons from acetate and transfer electrons to reduce Fe(III) with the HSs as the electron mediator. Furthermore, literatures showed some anaerobic respiratory bacteria in soils and sediments could use humic acid as the electron shuttles or donors to reduce nitrate and thus promote denitrification process^12–14^. However, since most of these researches were based on model microorganisms, it is not enough to predict the similar roles of HS in the microbial denitrification process for leachate, which is always treated by activated sludge (consists of mixed microorganisms)^15–18^.

It is widely accepted that the nitrogen oxide reductases, including nitrate reductase (NAR), nitrite reductase (NIR), nitric oxide reductase (NOR), and nitrous oxide reductase (N2OR), play important roles in microbial denitrification process (NO_3_ → NO_2_ → NO → N_2_O → N_2_)^19,20^. With the help of electron transport system, organic matters (especially the easy biodegradable organics) supply electrons (NADH) for these nitrogen oxide reductases to catalyze the reduction of nitrogen oxide. The literatures exhibited that the functional gene expressions involved in denitrification, carbon source metabolism, and electron transport system also influenced the denitrification process. Besides, the HS-oxidizing potential and the reduction rates of nitrogen oxide relate to the microbial community structure^21–23^. Therefore, the denitrification performance is affected by the above mentioned factors which need to be investigated.

The inherence of HS in leachate makes difficulties to allow the direct comparison of denitrification performance between the leachates with and without HS. These may be some reasons why the effects of HS on denitrification performance of leachate are still unknown. As the humic acid has been widely used as the model HS^24–26^, in our study, we used the model Shanghai Aladdin Humic Acid (SAHA) to investigate the effects of HS on denitrification performance for leachate with an anaerobic activated sludge process. The effects obtained from synthetic leachate were confirmed by adding extra SAHA into real leachate denitrification process. Finally, the mechanisms that HS affecting microbial denitrification for leachate were explored by several aspects: (1) microbial community structure shift, (2) the key gene expressions and activities of the key enzymes related to denitrification and carbon source metabolism, and (3) the electron transport system activity and reducing power generation.

## Materials and Methods

### Synthetic Wastewater and Landfill Leachate

A certain quantity of concentrated SAHA solution (500 mg/L) were added into a kind of medium (containing 2.94 g/L of CH_3_COONa, 2.88 g/L of KNO_3_, 0.5 mL/L of ‘P-water’, 2 mL/L ‘nutrient water’ and 0.5 mL/L ‘trace element water’). This resulted in a kind of synthetic wastewater consisting of 2000 mg/L of COD, 400 mg/L of nitrate-nitrogen and SAHA at the concentration 10 mg/L). In preliminary research (Fig. S3), we used 0, 5, 10 and 20 mg/L of SAHA to study the short-term effect of SAHA on the microbial denitrification of synthetic wastewater. For the short-term effects of 10 and 20 mg/L of SAHA on denitrification performance are almost the equally significant, 10 mg/L of SAHA was selected as the target concentration to study the long-term effects on denitrification performance. The pH was adjusted to 7.2 ± 0.1 by adding 4 mol/L NaOH or 4 mol/L HCl. The spectroscopic characteristics of SAHA, composition of ‘P-water’, ‘nutrient water’ and ‘trace element water’ were detailed in supporting information.

The landfill leachate was obtained from the effluent of nitrification reactor in a municipal composting field in Suzhou. Characteristics of the leachate are as follows: COD_Cr_ 2136 mg/L, BOD_5_ 1132 mg/L, NH_4_^+^-N 23 mg/L, NO_3_^−^-N 345 mg/L, NO_2_^−^-N 13 mg/L, total N 397 mg/L, alkalinity 47 mg/L, total P 9.8 mg/L and pH 7.3.

### Activated Sludge Acclimation

Activated sludge taken from Qingpu municipal wastewater treatment plant in Shanghai, was used as the seed sludge and acclimated in a sequencing batch reactors (SBR). The SBR was covered with aluminum foil to avoid the possible light-induced effects, maintained at 20 ± 1 °C, and worked with three 8-h-cycles per day. Each cycle consisted of 15 min influent, 5 h anaerobic and 5 min nitrogen gas stripping periods, followed by 1 h settling, 10 min decanting and 1.5 h idle periods. According to the literature, we prepared a synthetic medium which consisted of 0.6 g L^−1^ of CH_3_COONa, 0.72 g/L of KNO_3_, 0.5 mL/L of ‘P-water’, 2 mL/L ‘nutrient water’ and 0.5 mL/L ‘trace element water’. In the first 15 min of each cycle, activated sludge was fed with 3 L of the diluted medium which contained 400 mg/L of chemical oxygen demand (COD), 100 mg/L of nitrate-nitrogen. The influent pH was adjusted to 7.2 ± 0.1 by adding 4 mol/L NaOH or 4 mol/L HCl. After the nitrogen gas stripping period, 400 mL of sludge was wasted to keep the solids retention time (SRT) at approximately 10 days. The effluent concentrations of COD and NO_3_^−^-N in all SBRs were frequently determined until their removal reached relatively stable (approximately 60 days). *Thauera denitrificans* acclimation method was the same.

### Long-term Effects of SAHA on Denitrification

The experiments were conducted in serum bottles by exposing acclimated activated sludge to the SAHA contained synthetic wastewater. The acclimated activated sludge was obtained by centrifugation at 2000 g for 5 min and washed 3 times with 0.9% NaCl solution before being resuspended in the SAHA contained synthetic wastewater. The initial concentration of mixed liquid suspended solids (MLSS) was controlled at 150 mg/L. The effects of SAHA on denitrification were investigated by an additional cycle (20 h) after long-term exposure (30 d). Gas argon was purged into each bottle for 10 min to ensure the anaerobic condition. All bottles (set up in triplicate) were sealed and placed at constant temperature of 30 °C. The concentrations of NO_3_^−^-N, NO_2_^−^-N, N_2_O and key enzyme activities were measured during the experiments.

### Measurements of NO_3_^−^, NO_2_^−^ and N_2_O

This study used the spectrophotometric methods for the determinations of NO_3_^−^ and NO_2_^−^^27^. Briefly, 1 mL of sample was added in a 50-mL colorimetric tube with 49 mL of deionized (DI) water, followed by the addition of 1 mL of 1 mol/L hydrochloric acid solution. Then, the solution was shaken thoroughly, and the absorbance of the solution and standard (sodium nitrate) was measured spectrophotometrically at 220 and 275 nm. Finally, the absorbance at 275 nm was doubled and subtracted from that at 220 nm to give the corrected nitrate absorbance using for the calculation of the nitrate concentration. For the measurement of NO_2_^−^, 1 mL of sample was added in a 50-mL colorimetric tube, followed by the addition of 1 mL of Griess reagent (1% sulphanilamide, 2% H_3_PO_4_ and 0.1% naphthy-lethylenediamine dihydrochloride). The solution was immediately mixed by inversion and incubated at room temperature for 15 min. Then, the absorbance was determined at 540 nm, which was used to calculate the nitrite concentration. The direct microelectrode measurements of liquid-phase N_2_O concentrations were used in enzyme assays, and the microsensors were two-point calibrated using distilled water (zero point) and a freshly prepared 0.15 mmol/L N_2_O solution according to the manufacturer’s instructions (Unisense, Aarhus, Denmark).

### Enzymes Assays

The mixture was withdrawn at the end of additional cycles after long-term exposure, and washed 3 times with 0.01 mol/L phosphate buffer (pH 7.4) before measuring enzyme activities^28^. Then, the resuspended pellets were sonicated at 20 kHz and 4 °C for 5 min to break down cell structure of activated sludge. The debris was centrifuged at 12000 *g* and 4 °C for 10 min and the crude extracts in supernatant were obtained for measuring enzymatic activities. All enzymatic activities were based on protein content as measured according to the literature with bovine serum albumin as standard^29^, and the relative enzymatic activity was expressed as the ratio of the specific activity in exposed sludge to that in unexposed sludge. In all these measurements, the substrate was added only after a stable endogenous rate of change had been recorded to provide a reliable base-line. Supplementary Information listed the details of determination of enzyme activities including NAR, NIR, NOR, N2OR, acetyl-CoA synthetase (ACs), citrate synthase (CS), aconitate hydratase (AH), isocitrate dehydrogenase (IDH), α-Ketoglutrate dehydrogenase (KGDH), succinyl-CoA synthase (SCAs), succinate dehydrogenase (SDH), fumarate hydratase (FH), malate dehydrogenase (MDH), and isocitrate lyase (IL).

### DNA extraction, PCR and pyrosequencing

Sludge samples were the composite samples, which were taken from the long-term exposure experiment at the end of the one additional cycling, which contained synthetic wastewater. Sludge samples (500 μL) were centrifuged at 4000 rpm for 5 min at 4 °C, then the supernatant was removed and the condensed sludge was used for DNA extraction using the Genomic DNA Mini Preparation Kit with Spin Column (Beyotime Biotechnology, China). The purification of the extracted DNA was determined by 1% agarose gel electrophoresis. The DNA samples were stored at −20 °C before the next analyses.

The 16 S rDNA variable V3 region of extracted DNA was amplified with primer sets 338 F (5′-ACTCCTACGGGAGGCAGCAG-3′) and 806 R (5′-GGACTACHVGGGTWTCTAAT-3′). PCR amplification was carried out in a total volume of 20 μL containing 5 × FastPfu Buffer (4 μL), 2.5 mmol/L dNTPs (2 μL), 5 μmol/L Forward Primer (0.8 μL), 5 μmol/L Reverse Primer (0.8 μL), FastPfu Polymerase (0.4 μL), 10 ng of template DNA, BSA (0.2 μL), using ABI GeneAmp 9700. The amplification program consisted of an initial denaturation step at 95 °C for 3 min, 27 cycles of amplification (at 95 °C for 30 s, 55 °C for 30 s, and 72 °C for 45 s), extension at 72 °C, and followed by a final annealing at 10 °C for termination. The PCR products were pooled and purified using the AxyPrepDNA Gel Extraction Kit (AXYGEN, USA). Finally, the DNA library was constructed and run on the Miseq Illumina at the Beijing Genome Institute (Shenzhen, China).

### RNA-Seq

The RNA-Seq analysis was used to determine the transcriptional profiling of pure *Thauera* in the long-term absence (the control) and presence of 10 mg/L SAHA. Briefly, the *Thauera* cells were harvested from the additional cycle experiment at 20 h, followed by centrifugation at 10 000 rpm for 10 min at 4 °C, and then lysed in TRIzol reagent (Invitrogen) for extraction of total RNA. To avoid genomic DNA contamination, the extracted RNA was treated with DNase I (Ambion) according to the manufacturer’s protocol. Thereafter, mRNA was isolated from the DNA-free total RNA using the MICROBExpress Bacterial mRNA Enrichment Kit (Ambion), and prepared for Illumina sequencing using the mRNA-Seq Sample Preparation Kit (Illumina) according to manufacturer’s instructions. The RNASeq libraries were finally sequenced using an Illumina HiSeq. 2000.

The next generation sequencing (NGS) quality control (QC) toolkit v2.2.1 was used to filter the raw reads^30^ by removing the reads with (1) sequence adapters, (2) more than 5% “N” bases, and (3) more than 50% QA ≤ 15 bases. The obtained clean reads were aligned to the reference genome using SOAP^31,32^ and no more than 3 mismatches were allowed in the alignment for each read. The gene expression level was calculated using the RPKM (reads per kilobase of exon region per million mappable reads) method^33^, and the differentially expressed genes were identified based on the criteria: absolute fold change > 2 and false discovery rate (FDR) < 0.05. Gene ontology (GO) and Kyoto Encyclopedia of Genes and Genomes (KEGG) analyses were performed using Blast2GO using default annotation parameters. Interactive Pathways (ipath) analysis was carried out via interactive pathways explorer v2 (http://pathways.embl.de/).

### Other Methods

NADH/NAD^+^ assay and electron transport system activity (ETSA) were documented in the Supplementary Information.

### Statistical analysis

All tests in this study were performed in triplicate. An analysis of variance was used to test the significance of results, and p < 0.05 was considered to be statistically significant.

## Results and Discussion

### Effects of SAHA on Microbial Denitrification

**Figure 1:**
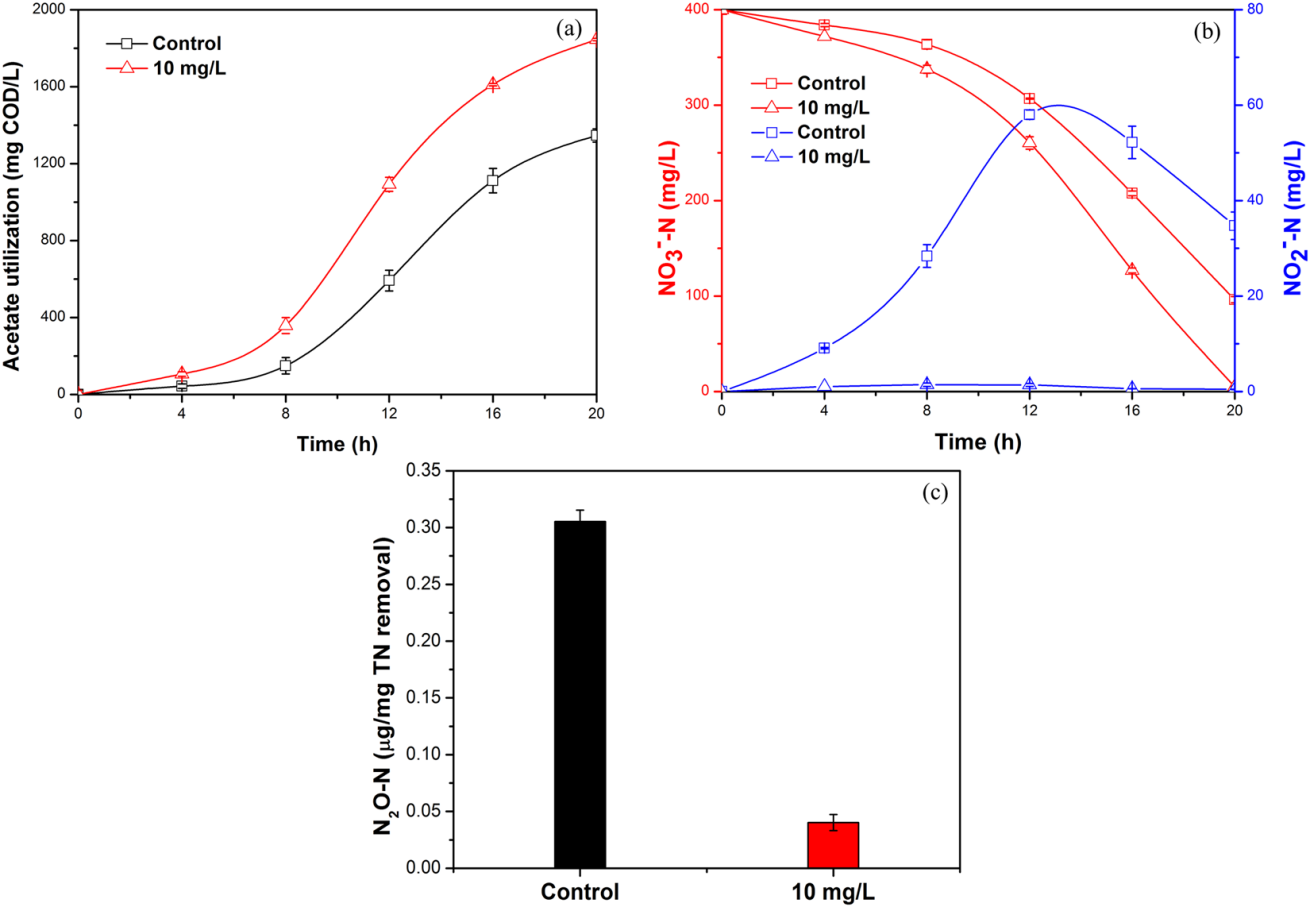
Effects of 10 mg/L SAHA on the changes of COD, NO_3_^−^-N, NO_2_^−^-N and N_2_O during one cycle after long-term exposure for synthetic wastewater treatment. Error bars represent standard deviations of triplicate measurements.

Synthetic wastewater was initially used as the study medium. As an important component in landfill leachate, HS can chronically influence the microbial denitrification process. Therefore, long-term exposure of activated sludge to SAHA was conducted to investigate the long-term effects of SAHA on denitrification of activated sludge. One additional cycle was operated after long-term exposure to 10 mg/L SAHA for 30 days. Figure 1a presents the acetate utilization resulted from 10 mg/L SAHA was higher than that from control (1845 versus 1346 mg COD/L). As seen from Fig. 1b, the final nitrate concentration at 20 h in the presence of 10 mg/L SAHA was much lower than that in control (5.2 versus 96.2 mg/L). More importantly, nearly no nitrite was accumulated in whole cycle with the presence of 10 mg/L SAHA, which was not achieved from the long-term operated control. Similarly, total N_2_O generation in 20 h with the long-term presence and absence of SAHA were 0.040 and 0.305 (μg/mg TN removal), respectively. It means long-term exposure to SAHA decreased 86.9% N_2_O generation compared with the control experiment. This positively affects the environment, because N_2_O was deemed to result in global warming 300 times higher than carbon dioxide^34^ and severely destroy ozone instratosphere^35^.

**Figure 2:**
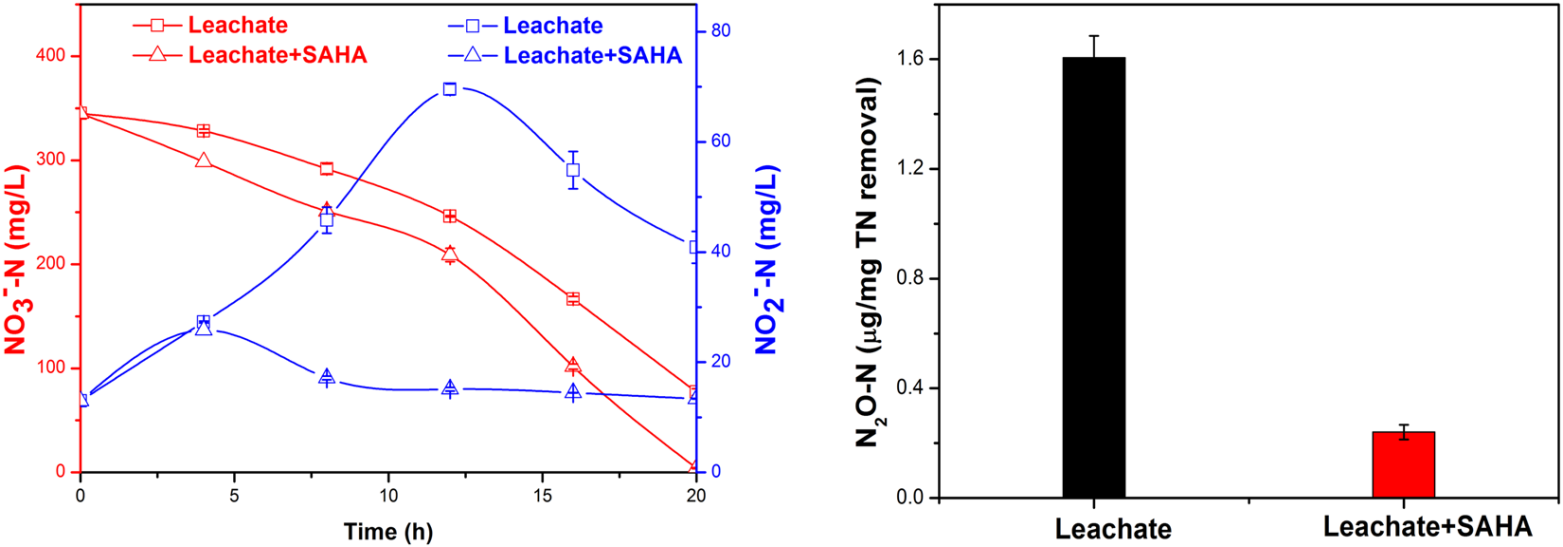
Long-term effects of SAHA on the denitrification for real leachate. Error bars represent standard deviations of triplicate measurements.

SAHA was observed to enhance microbial denitrification for synthetic wastewater. Therefore, 10 mg/L SAHA was further used to investigate the effects of HS on microbial denitrification for real leachate. As shown in Fig. 2, the final NO_3_^−^-N, and NO_2_^−^-N and N_2_O concentrations remarkably decreased from 77.2 to 4.2 mg/L, 40.9 to 13.4 mg/L, 67.6 to 0.6 mg/L and 1.61 to 0.24 μg/mg TN removal, respectively, after the addition of SAHA. Even more, NO_2_^−^-N accumulation peak for leachate + SAHA reached earlier and smaller than that for leachate, which suggests the presence of SAHA accelerated nitrate and nitrite reduction. These results obviously confirmed the noticeable promotion of SAHA to the denitrification for real landfill leachate.

### Effects of SAHA on Bacterial Community Shift in Activated Sludge

**Figure 3:**
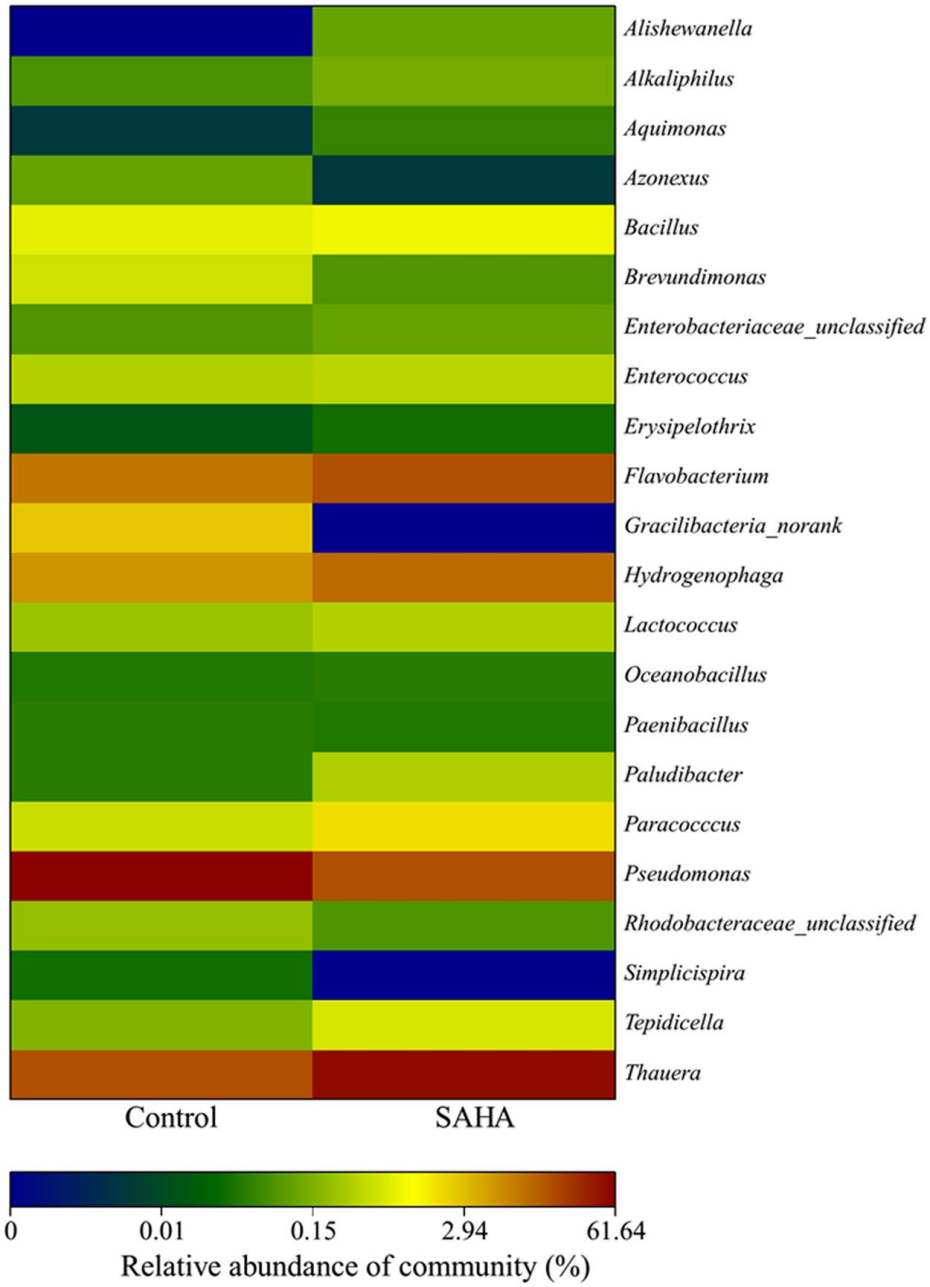
Long-term effects of SAHA on genus level distributions of activated sludge.

The literature reported that the composition of bacterial community would significantly determine the function of activated sludge which used in wastewater treatment. Herein, we used high-throughput pyrosequencing technology to analyze the bacterial communities of the activated sludge after 30 d experiments’ running with the absence and presence of SAHA. As shown in Fig. 3, the bacterial diversity of the activated sludge in the control experiment was not significantly different from that after long-term exposure to SAHA. The bacteria involved in denitrification, included *Bacillus* ^36^, *Paracoccus* ^20^, *Pseudomonas* ^37^ and *Thauera* ^38,39^, were observed in the two experiments. However, the proportions of these microorganisms accounted in the activated sludge were altered after long-term presence of SAHA. The proportions of *Bacillus* and *Paracoccus*, slightly increased to 1.1% and 1.8% in the presence of SAHA, compared to 0.8% and 0.5% in the control, respectively. Furthermore, *Thauera* was greatly enriched and dominated in the SAHA effected activated sludge compared to the control (48.5% versus 17.4%). In contrast, *Pseudomonas* accounted for 61.6% and dominated in the control system, while constituted only 17.5% in the presence of SAHA.

Liu *et al*.^38^ found the *Thauera* extracted from anaerobic activated sludge had a strong ability to reduce nitrite, because nitrite hardly accumulated during the denitrification. In addition, *Thauera* is known to be able to biodegrade organic matters during denitrification, which has been frequently detected in wastewater treatment bioreactors^38,39^. In our study, the predominance of *Thauera* may be one important reason for the improved acetate utilization, the nearly absence of nitrite and enhanced NIR activity after long-term exposure to SAHA. In comparison, a previous study reported that nitrite accumulated when acetate served as the only carbon and electron source for *Pseudomonas*, which could be ascribed to the larger rate of nitrate reduction than nitrite reduction and the competition between nitrate and nitrite reduction pathways for electrons^40^. This study indicated the electron flow to nitrite reductases (NIR) was overwhelmed by that to nitrate reductases (NAR) during acetate oxidation in *Pseudomonas* cells. In this case, nitrite reduction was the rate limiting step in denitrification process. In addition, nitrous oxide accumulation was found high when *Pseudomonas* was denitrifying under anaerobic conditions^41^. However, humic acid can be reduced by *Thauera* ^42^ instead of *Pseudomonas* ^43^, thus acted as electron donor and shuttle to accelerate electron transport and relieve the inhibition of nitrate to nitrite reduction. Therefore, long-term exposure to SAHA can selectively promote *Thauera* abundance and the intrinsic electron transport of nitrite reduction and finally enhanced the denitrification performance.

**Figure 4:**
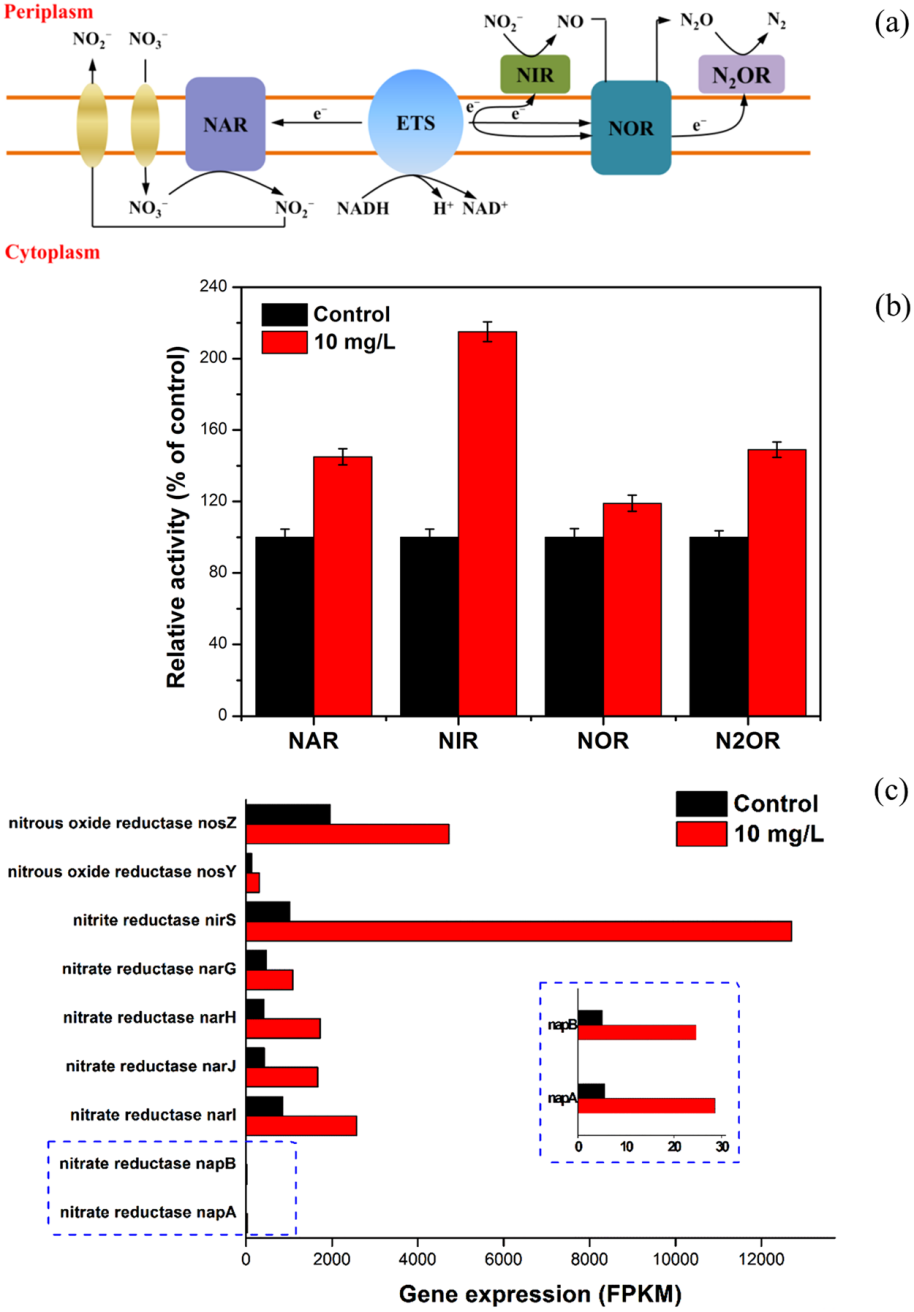
Schematic denitrification and electron transport pathway in denitrification (**a**), relative activities of key denitrifying enzymes after long-term exposure to SAHA (**b**), key gene expressions involved in denitrification enzymes in *Thauera denitrificans* (**c**). Error bars represent standard deviations of triplicate tests.

The schematic denitrification pathway (Fig. 4a) clearly presents NADH acts as an immediate electron source for denitrification and electron transport system affects denitrification performance. In the presence of SAHA, the ETSA value of activated sludge was obviously higher than that of control (0.180 versus 0.136 μg O_2_·g^−1^ protein·min^−1^, see SI, Fig. S4). Electrons were directed to reduce NO_3_^−^, NO_2_^−^, NO, and N_2_O by the catalysis of NAR, NIR, NOR and N2OR, respectively. The four key denitrification enzymes, to a major extent, determine the denitrification performance. Hence, in this study, the effects of long-term presence of SAHA on these key denitrification enzymes were evaluated. As exhibited in Fig. 4b, the activities of NAR, NIR, NOR and N2OR greatly increased to 145%, 215%, 119% and 149% of the control after long-term exposed to 10 mg/L SAHA, respectively. The drastic increase of NIR with exposure to SAHA may be a vital reason to result in almost absence of NO_2_^−^ in the effluent. According to previous research, NO_2_^−^ or its derived HNO_2_ accumulation would inhibit the denitrifying reaction of N_2_O to N_2_ in the biological nitrogen removal process^44^. Therefore, the reduced levels of nitrite would significantly promote the transformation of N_2_O to environment-friendly N_2_ and lead to less N_2_O accumulation. In addition, the level of the N2OR activity/NOR activity ratio was observed to influence N_2_O accumulation in literature^45,46^. As observed from Fig. 4b, long-term effect of SAHA augmented the N2OR activity/NOR activity ratio to 1.25 times of the control, which may be another important reason for N_2_O accumulation.

Microbial community analysis shows that the predominance of *Thauera* made great contribution to the denitrification performance. To further understand the behind mechanism, we additionally incubated pure *Thauera* cells in the long-term absence and existence of SAHA. The acclimation procedure and effect experiment method were consistent with that of activated sludge. RNA-Seq analysis was used to determine and compare the transcriptional profiling of *Thauera denitrificans* with and without SAHA.

It is well-known that NAR, NIR, NOR and N2OR are encoded by the gene groups of *nar*GHJI (encoding respiratory nitrate reductase), *nap*ABC (encoding periplasmic nitrate reductase), *nir*SECF, *nor*CBQDEF, and *nos*RZD, respectively^47^. Figure 4c illustrates that the gene expressions associated with NAR, NIR and N2OR synthesis were substantially up-regulated in the presence of SAHA, while there were no significant differences between the NOR encoding gene expressions in the absence and presence of SAHA. The increased gene expressions may explain the dominance of *Thauera*, enhanced activities of key denitrifying enzymes and the improved denitrification performance. In particular, the up-regulated folds for narI, napA, nirS, and nosZ were 1.6, 2.4, 3.6, and 1.3, respectively. Obviously, the up-regulated fold for nir operon was much greater than the other operons, which may be an important reason for the observed nearly complete removal of nitrite.

In addition, according to a previous literature^48^, heme c and heme d1 located at the catalytic center of NIR and Cu ions served as the catalytic center of N2OR. Meanwhile, it has been reported that the transcriptional genes nirF, nirG, nirH, and nirT were responsible for the synthesis of heme c and heme d1^49,50^, and the gene nosL was involved in the synthesis of Cu-catalytic center^51^. In our study, these gene expressions were also up-regulated (Table S3, SI) and beneficial to improve NIR and N2OR activities, thus the increased nitrite removal and decreased N_2_O accumulation.

### Effects of SAHA on acetate metabolism

**Figure 5:**
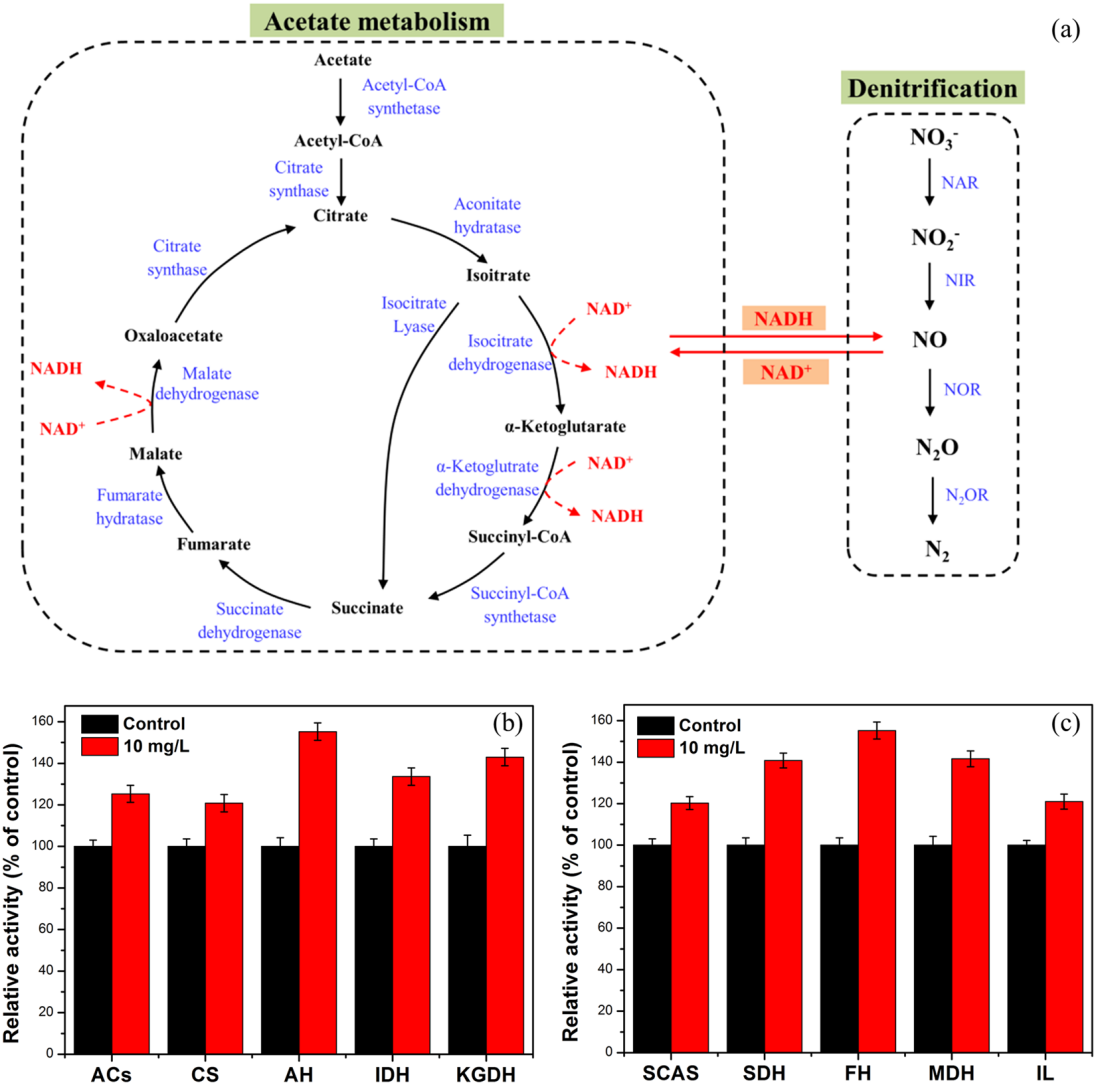
Long-term effects of SAHA on the acetate metabolism of activated sludge. (**a**) Schematic metabolic pathways of acetate in activated sludge. (**b**) Relative activities of ACs, CS, AH, IDH, KGDH. (**c**) Relative activities of SCAS, SDH, FH, MDH and IL. Error bars represent standard deviations of triplicate measurements.

During the denitrification process, denitrifying microorganisms metabolize carbon sources to generate electron donor NADH which supplies the electrons for nitrate reduction^52^. Therefore, the acetate metabolism involved in NADH generation, including TCA cycle, was evaluated in this study. Long-term exposure to SAHA caused the sharp increase (p < 0.05) of key enzymes activities related to acetate metabolism and NADH generation (IDH, KGDH and MDH) (Fig. 5b and c). This is one important reason for the enhanced acetate degradation and a significant increase of NADH/NAD^+^ ratio by the long-term exposure of SAHA (see SI, Fig. S5). Compared with the control, the presence of SAHA enlarged the NADH generation, which could supply more electrons for the denitrifying enzymes reducing nitrogen oxides.

**Figure 6:**
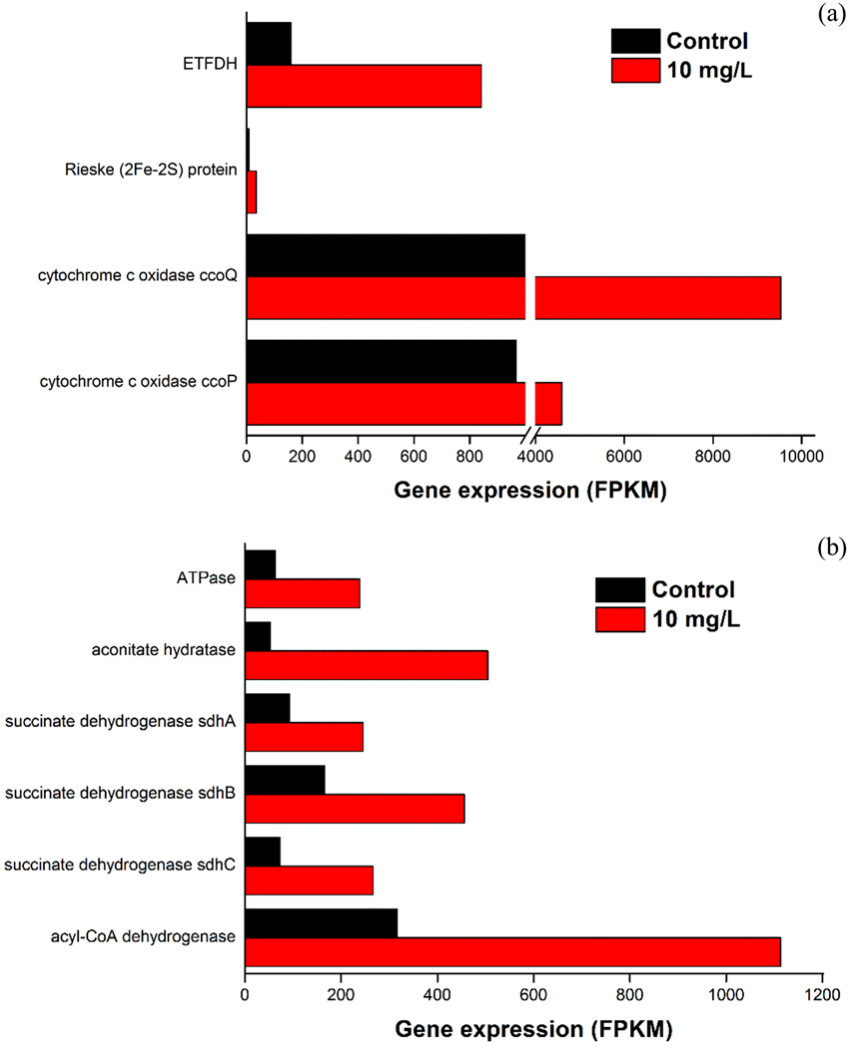
Long-term effects of SAHA on key gene expressions involved in electron transport (**a**) and key acetate metabolic enzymes (**b**) in *Thauera denitrificans*.

In most denitrifying bacteria, the electron flow directs from NADH to nitrogen oxide reductases (NAR, NIR, NOR and N2OR) through the respiratory chain step by step^53^. Hence, the transcriptional levels of key genes related to electron transport system were characterized. As shown in Fig. 6a, the gene expressions of these essential complexes, such as NADH dehydrogenase, electron-transporting-flavoprotein dehydrogenase (ETFDH), succinate dehydrogenase and cytochrome c, were significantly up-regulated in the presence of SAHA. Specifically, flavin mononucleotide and flavin adenine dinucleotide are two kinds of flavoprotein which involved in NADH and succinate dehydrogenation, respectively^54^. Our data present that SAHA promoted ETSA for the increase of the related gene expressions. In the literature, cytochrome c was found to function vitally in nitrite-reducing cells which incubated with acetate^53^. In our study, gene expression related to cytochrome c was enhanced the most in these key gene expressions involved in electron transport system. This may be an important reason for the great promotion not only in nitrate reduction but also in nitrite reduction when *Thauera* dominated in activated sludge after long-term exposed to SAHA. The gene expressions of several key enzymes associated with acetate metabolism, such as aconitate hydratase and the already-mentioned succinate dehydrogenase, were up-regulated in the presence of SAHA. Meanwhile, the activities of the two enzymes in the activated sludge presented with SAHA were tremendously enhanced (Fig. 5). The results showed the positive effect of SAHA on acetate biodegradation was ascribed to the significant increase of gene expressions and enzyme activities involved in acetate metabolism.

## Conclusions

This study demonstrated that the presence of HSs benefited the denitrification process for landfill leachate. As activated sludge exposed to the model HS (SAHA), the community structure was shifted and the resulted dominance of *Thauera* was one important reason for the enhanced denitrification performance. It was observed that SAHA obviously increased the catalytic activities of the key enzymes involved in carbon source metabolism and denitrification process. The improved electron transport system activity and reducing power (NADH) were beneficial to the nitrogen oxide reducing reaction which occurred during denitrification process. Besides, RNA-Seq analysis showed these corresponding gene expressions were also largely upregulated.

## Electronic supplementary material

Supplementary information_Inherent humic substance promotes microbial denitrification of landfill leachate via shifting bacterial community, improving enzyme activity and up-regulating gene
