# Supplementary material for: Inherent humic substance promotes microbial denitrification of landfill leachate via shifting bacterial community, improving enzyme activity and up-regulating gene
Source: Sci Rep. 2017 Sep 22;7:12215. doi: 10.1038/s41598-017-12565-3 (PMC5610334; doi:10.1038/s41598-017-12565-3)
Supplement: Supplementary file 1 — Supplementary information_Inherent humic substance promotes microbial denitrification of landfill leachate via shifting bacterial community, improving enzyme activity and up-regulating gene [file 41598_2017_12565_MOESM1_ESM.doc]

# Supplementary Information

**Inherent humic substance promotes microbial denitrification of landfill leachate via shifting bacterial community, improving enzyme activity and up-regulating gene**

Shanshan Dong, Mu Li, Yinguang Chen*

(*State key laboratory of pollution control and Resource reuse, College of Environmental Science and Engineering, Tongji University,* *1239 Siping Road, Shanghai 200092, China*)

* Corresponding author Tel.: +86 21 65981263; fax: +86 21 65986313

E-mail address: [yg2chen@yahoo.com](mailto:yg2chen@yahoo.com)

Journal: Scientific Reports

Number of pages: 18

Number of tables: 3

Number of figures: 5

# Materials and Methods

**‘P-water’, ‘nutrient water’ and ‘trace element water’**

The ‘P-water’ consisted of (g/L): 65.13 KH_2_PO_4_ and 97.26 K_2_HPO_4_·3H_2_O. The ‘nutrient water’ contained (g/L): 133.75 NH_4_Cl. The ‘trace element solution’ contained (g/L): 1.50 FeCl_3_·6H_2_O, 0.03 CuSO_4_·5H_2_O, 0.12 MnCl_2_·4H_2_O, 0.06 Na_2_MoO_4_·2H_2_O, 0.12 ZnSO_4_·7H_2_O, 0.15 CoCl_2_·6H_2_O, 0.18 KI, 0.15 H_3_BO_3_ and 10 ethylenediamine tetraacetic acid.

**Fourier Transform Infrared (FTIR), Fluorescence and Solid-state ^13^C NMR Analysis of SAHA.**

**FTIR.** The freeze-dried SAHA samples were prepared by mixturing of 1 mg of freeze-dried sample and 300 mg of IR-grade potassium bromide (KBr), then grounded and homogenized to reduce light scatter. After that, the subsample was compressed in chip module under the pressure of approximately 20 000 psi to form a KBr window. The FTIR was set to scan from 4000 to 400 cm^−1^ with a Nicolet 5700 FTIR spectrometer. The spectra of each sample were acquired by deduction of the background spectra (pure KBr) from the spectra of KBr-mixed sample.

**Flurorescence.** Fluorescence measurements were conducted using a luminescence spectrometry (Fluoromax-4 Spectrofluorometer, HORIBA Scientific, France). To obtain the spectra of fluorescence excitation emission matrix (EEM), the excitation wavelengths were increased from 250 to 550 nm at 5 nm increments, and the emission wavelengths were detected from 350 to 650 nm every 5 nm. 50 mg C/L (DOC) SAHA samples were measured under pH 7.0 ± 0.1 (the pH adjusting process was under N_2_ atmosphere), and the ionic strength was 0.01 mol/L KCl.

**^13^C NMR.** The solid-state ^13^C-NMR spectra of SAHA were acquired at 100.61 MHz on a Bruker Avance 400 NMR spectrometer. A standard 4.0 mm magic angle spin (MAS) double-resonance probe head was used in the experiments and spinning speed of the rotor was set at 5 kHz. The acquisition time and delay time were 14.62 ms and 15 s, respectively. The ^13^C NMR spectra were normalized by setting their total area as 100%.

## Determination of Enzyme Activities

**NAR, NIR, NOR, and N2OR.** For determining the activities of denitrification reductases (NAR, NIR, NOR, and N2OR), the assay mixture (1.7 mL) contained 10 mmol/L PBS buffer (pH 7.4), 1 mmol/L methyl viologen, 5 mmol/L Na_2_S_2_O_4_, and 5 mmol/L reaction electron acceptor (KNO_3_, NaNO_2_, NO, or N_2_O) [^1^](#_ENREF_1). All the above substances were diluted from stock solution and saturated solutions of NO and N_2_O. The saturated solutions (2.0 mmol/L for NO and 25 mmol/L for N_2_O) were prepared by purging pure NO or N_2_O gas into Milli-Q water continuously for 5 min. It should be concerned that all the operations about NO were under anaerobic conditions or Ar protection for NO being easily oxidized by oxygen. The reaction was started by adding 0.3 mL crude cell extracts into the assay mixture. Then the mixture was immediately settled in a 30 °C incubator, and the data were collected every 10 min. The concentration of NO_2_^−^-N, NO, or N_2_O was determined, and the enzyme activity was calculated. In detail, the increased or decreased NO_2_^−^-N concentration was detected by a spectrophotometer for NAR and NIR measurements, and the consumptions of NO or N_2_O in mixture were recorded by corresponding microsensors (Unisense, Denmark) for determination of NOR and N2OR. The activities of NAR and NIR were expressed respectively as the production and reduction of μmol nitrite min^−1^ mg^−1^ protein. For NOR and N2OR, the units of enzymatic activities were the consumptions of μmol nitric oxide min^−1^ mg^−1^ protein) and μmol nitrous oxide min^−1^ mg^−1^ protein, respectively.

**ACs.** The activity of acetyl-CoA synthetase (ACs) was measured using the hydroxylamine assay with minor modification from the literature [^2^](#_ENREF_2). This procedure measures colorimetrically acetylhydroxamate formation by ACs from ATP, coenzyme A (CoA), acetate, and hydroxylamine. After 5 min of preincubation at 35 °C, 500 μL of crude extracts was added into the medium which contained 50 mmol/L hydroxylamine (pre-neutralized by KOH), 50 mmol/L Tris-HCl (pH 8.0), 20 mmol/L sodium acetate, 10 mmol/L MgCl_2_, 10 mmol/L ATP, 2 mmol/L dithiothreitol (DTT) and 1 mmol/L CoA. The pH of the reaction mixture was controlled at an optimum pH (8.0) because it strongly affected the activity. After incubation for 10 min, the reaction was terminated by the addition of ferric chloride reagent (0.5 ml of 10% (w/v) FeCl_3_ in 2 M HCl, 3.3% (w/v) trichloroacetic acid (TCA)). The tubes were centrifuged at 12000 g for 5 min to remove turbidity, and the color generated was measured at 540 nm. Samples without crude extracts served as a blank. The ACs activity was given as μmol hydroxamate min^−1^ mg^−1^ protein.

**CS.** Citrate synthase (CS) activity was assayed similar to a method in the literature [^3^](#_ENREF_3). The 2 mL of assay medium contained 50 mmol/L Tris-HCl (7.2), 0.2 mmol/L 5,5'-dithiobis-(2-nitrobenzoic acid) (DTNB), 0.1 mmol/L acetyl-CoA, 0.5 mmol/L oxaloacetate and 50 μL of crude extracts. Citrate synthase activity was assayed by the absorbance at 412 nm at 25 ^o^C and was given as the consumption of μmol acetyl-CoA min^−1^ mg^−1^ protein.

**AH.** Aconitate hydratase (AH) activity was determined by a modified method [^4^](#_ENREF_4). The reaction mixture contained the 100 mmol/L potassium phosphate-sodium phosphate buffer (pH 7.4), with 50 mmol/L sodium citrate as substrate; the increase in absorbance at 240 nm at 25 ^o^C was recorded. Aconitate hydratase activity was given as the increase of μmol D, L-isocitrate min^−1^ mg^−1^ protein.

**IDH.** Isocitrate dehydrogenase (IDH) was determined in 50 mmol/L potassium phosphate buffer (pH 6.85), containing 5 mmol/L MgSO4, 0.2 mmol/L NADP, 10 mmol/L D, L-isocitrate, and 200 μL of crude extracts; the increase in absorbance at 340 nm was at 25 ^o^C was recorded. IDH activity was given as the production of μmol NADH min^−1^ mg^−1^ protein.

**KGDH.** α-Ketoglutrate dehydrogenase (KGDH) activity was measured according to a method in the literature [^5^](#_ENREF_5). 600 μL of crude extracts was suspended in a 2 mL of solution containing 0.6 mmol/L NAD^+^, 0.1 mmol/L thiamine pyrophosphate (TPP), 0.08 mmol/L CoA and 4 mmol/L ketoglutarate. Enzyme activity was assayed by measuring the production of NADH at 340 nm at 25 ^o^C.

**SCAs.** The assay of succinyl-CoA synthase (SCAs) activity was performed at 30 °C using a modification of the procedure of the literature [^6^](#_ENREF_6). 500 μL of crude extracts was added in a 2 mL of solution containing 50 mmol/L potassium phosphate (pH 7.2), 10 mmol/L MgCl_2_, 0.2 mmol/L succinyl-CoA, 2 mmol/L ADP, and 0.2 mmol/L DTNB. The reaction was initiated by adding succinyl-CoA and DTNB in quick succession. Rates were corrected by subtracting the rate observed when ADP was deleted. The SCAs activity was measured by the production of μmol thionitrobenzoate min^−1^ mg^−1^ protein at 412 nm.

**SDH.** Succinate dehydrogenase (SDH) activity was measured spectrophotometrically according to [the](http://www.sciencedirect.com/science/article/pii/S0308814614007699#b0050) literature [^7^](#_ENREF_7), by oxidation of succinate with potassium ferricyanide acting as the artificial electron acceptor. The reaction mixture contained 890 μL of phosphate buffer (0.2 mol/L, pH 7.8), 50 μL of sodium succinate (0.6 mol/L), 10 μL of potassium ferricyanide (0.03 mol/L), and 50 μL of crude extracts. The SDH activity was followed by absorbance change at 420 nm for 2 min and given as the consumption of μmol potassium ferricyanide min^−1^ mg^−1^ protein.

**FH.** Fumarate hydratase (FH) was determined in a medium which contained 60 mmol/L potassium phosphate-sodium phosphate buffer, pH 7.4, 200 μL of crude extracts, with 6.6 mmol/L sodium L-malate as substrate; the increase in absorbance at 240 nm was recorded [^4^](#_ENREF_4).

**MDH.** Malate dehydrogenase (MDH) was determined in a medium which contained was determined in a medium which contained 50 mmol/L potassium phosphate-sodium phosphate buffer, pH 7.4, 1 mmol/L oxaloacetate, 0.2 mmol/L NADH, 100 μL of crude extracts. The increase in absorbance at 340 nm was at 25 ^o^C was recorded. Malate dehydrogenase activity was given as the production of μmol NADH min^−1^ mg^−1^ protein.

**IL.** Isocitrate lyase (IL) was assayed spectrophotometrically at 324 nm, according to the rate of formation of glyoxylate phenylhydrazone in the presence of isocitrate and phenylhydrazine [^8^](#_ENREF_8). The reaction mixture contained 50 mmol/L potassium phosphate-sodium phosphate buffer, pH 7.4, 5 mmol/L MgCl_2_, 0.5 mmol/L ethylenediaminetetraacetic acid (EDTA), 6 mmol/L phenylhydrazine-HCl, 2 mmol/L D,L-isocitrate, and 300 μL of crude extracts. One unit of enzyme activity is denoted as the production of μmol glyoxylate phenylhydrazone min^−1^ mg^−1^ protein.

**NADH/NAD^+^ Assay.** The intracellular reduction equivalent (NADH) level was detected according to the literature [^9^](#_ENREF_9). Amount of 1 mL of the samples was collected at 20 h and centrifuged at 12000 g for 5 min. After removing the supernatant, 0.2 mol/L NaOH was added to re-suspend the pellets for NADH extraction. The samples were bathed at 50 °C for 10 min afterwards and then cooled down to 0 °C by ice. Thereafter, the extracts were neutralized by adding 300 μL of 0.1 mol/L HCl (for NADH extraction) drop-wise while vortexing. Supernatants were obtained by centrifugation at 15000 g for 5 min and transferred to new tubes for measurement immediately. The intracellular NADH concentrations were determined by the enzymatic cycling assay. The mixture of cycling assay consisted of equal volumes of 1.0 mol/L bicine buffer (pH 8.0), ethanol, 40 mmol/L EDTA (pH 8.0), 4.2 mmol/L thiazolyl blue (MTT), and twice the volume of 16.6 mM phenazine ethosulfate (PES), which was then incubated at 30 °C for 10 min. The reaction mixture was prepared as follows: 50 μL neutralized cell extract, 0.3 mL distilled water, and 0.6 mL reagent mixture. The reaction was started by adding 50 μL of alcohol dehydrogenase (ADH, 500 U/mL). The absorbance at 570 nm was checked every 30 s for 5 min at 30 °C. The concentration of NADH was calibrated with standard solutions of NADH, and the final NADH level was calculated as per unit protein.

**Electron Transport System Activity (ETSA) Assay.**

The electron transport system activity of activated sludge was determined by reducing 2-(p-iodophenyl)-3-(p-nitrophenyl)-5-phenyl tetrazolium chloride (INT, a kind of exogenous electron acceptor) to formazan (INF), which was modified from the literature [^10^](#_ENREF_10). After long-term exposure, the activated sludge was harvested by centrifugation at 5000 g for 15 min, rinsed twice with phosphate buffered saline (PBS, 50 mmol/L, pH 7.4) buffer, and then resuspended in 50 mmol/L PBS. Next, 200 μL of INT (0.5%) and 0.2 mg NADH were added into 1 mL of bacterial culture harvested from one of the tests. The mixture was subsequently incubated at 30 °C for 30 min in the dark, after which 100 μL of formaldehyde were added to terminate the reaction. The samples were subsequently centrifuged at 10 000 g for 3 min to collect the cells. Next, 500 μL of 96% methanol were used to extract the INF from the activated sludge twice, after which the mixed INF extract was measured spectrophotometrically at 490 nm against a solvent blank. The ETSA was calculated according to the following formula:

$ETSA\left(\mu gO_{2}\cdot g^{-1}\cdot protein\cdot min^{-1}\right)=\frac{ABS_{490}}{15.9}\frac{V_{1}}{V_{0}t}\frac{32}{2}\frac{1}{m}$ (1)

where, ABS_490_ is the sample absorbance, 15.9 is the specific absorptivity of INT-formazan, V_0_ and V_1_ are the initial volume of activated sludge and the total volume of methanol (mL), t is the incubation time (min), 32/2 is the constant for transformation of μmol INT-formazan to μg O_2_, and m is the protein concentration per milliliter of activated sludge (mg protein mL^−1^ activated sludge).

**Table S1**. Distribution of different carbons in SAHA calculated from ^13^C NMR spectrum. ^a^

| ^13^C-NMR | SAHA(%) |
| --- | --- |
| Aliphatic Carbon | 53.7 |
| Acetal Carbon | 12.3 |
| Aromatic Carbon | 19.9 |
| Carboxyl Carbon | 9.7 |
| Carbonyl Carbon | 4.4 |
| ^a^ The percentage peak areas of individual peaks were calculated by dividing their areas by the total spectral peak area of the sample. | |

**Table S2.** Summary of the clean reads mapped to the reference genomes and genes of *Thauera denitrificans* in the long-term absence (Control) and presence of 10 mg/L SAHA.

|  | | Control | | 10 mg/L SAHA | |
| --- | --- | --- | --- | --- | --- |
|  |  | Number of reads | Percentage | Number of reads | Percentage |
| Total number of high-quality reads | | 53481958 | 100% | 58422396 | 100% |
| Total basepairs of high-quality reads | | 7469030812 | 100% | 7469030812 | 100% |
| Map to Genome | Total mapped reads | 51088284 | 95.52% | 54494530 | 93.28% |
|  | Unique matches | 48432152 | 90.56% | 50973806 | 87.25% |
|  | Multi-postion matches | 2656132 | 4.97% | 3520724 | 6.03% |
|  | Total unmapped roads | 2393674 | 4.48% | 3927866.00% | 6.72% |
| Map to Gene | Total mapped reads | 38274506 | 71.57% | 41632162 | 71.26% |
|  | Unique matches | 34736932 | 64.95% | 35380938 | 60.56% |
|  | Multi-postion matches | 3537574 | 6.62% | 6251224 | 10.70% |
|  | Total unmapped roads | 15207452 | 28.43% | 16790234 | 28.74% |

**Table S3.** Key up- and down-regulated genes of *Thauera denitrificans* exposed to 10 mg/L SAHA for long-term.

| Gene ID | log_2_(SAHA/Control) | FDR | Gene description |
| --- | --- | --- | --- |
| NC_008687.1: c249529-252093 | 3.34 | 0.0001 | aconitate hydratase |
| NC_008686.1: c1101981-1102979 | 2.57 | 0.0005 | ATPase |
| NC_008686.1: 541157-541549 | 1.93 | 0.0001 | succinate dehydrogenase, cytochrome b subunit |
| NC_008686.1: 544520-545299 | 1.26 | 0.0001 | succinate dehydrogenase, iron-sulfur subunit |
| NC_008686.1: 541965-543767 | 1.45 | 0.0001 | succinate dehydrogenase, flavoprotein subunit |
| NC_008688.1: c493422-494525 | 1.87 | 0.0001 | pyruvate dehydrogenase E2 component |
| NC_008688.1: c494527-495534 | 1.78 | 0.0001 | pyruvate dehydrogenase E1 component subunit beta |
| NC_008688.1: c495557-496534 | 1.96 | 0.0001 | pyruvate dehydrogenase E1 component subunit alpha |
| NC_008688.1: c616216-618018 | 3.34 | 0.0001 | acetoacetyl-CoA reductase |
| NC_008686.1: 2507025-2508179 | 2.51 | 0.0001 | protein nirF cytochrome d1, heme region |
| NC_008686.1: 2509150-2509623 | 2.74 | 0.0001 | protein nirG, transcriptional regulator |
| NC_008686.1: 2508182-2509153 | 2.29 | 0.0001 | protein nirH, transcriptional regulator |
| NC_008686.1: 2509620-2510102 | 2.46 | 0.0001 | protein nirD, transcriptional regulator |
| NC_008688.1: 210265-210978 | 2.32 | 0.0001 | cytochrome C, nirT, transcriptional regulator |
| NC_008686.1: c2250693-2251220 | 1.78 | 0.0001 | NADH dehydrogenase, subunit B |
| NC_008686.1: c396309-397955 | 2.56 | 0.0001 | electron-transferring-flavoprotein dehydrogenase |
| NC_008686.1: 1842731-1844188 | 1.96 | 0.0001 | 4Fe-4S ferredoxin protein |
| NC_008686.1: c396309-397955 | 3.99 | 0.001 | Rieske (2Fe-2S) domain-containing protein |
| NC_008687.1: c1395758-1397275 | 1.95 | 0.0001 | nitrate reductase, subunit beta, narH |
| NC_008687.1: c1394356-1395057 | 1.65 | 0.0001 | nitrate reductase, subunit gamma, narI |
| NC_008687.1: c1397272-1401036 | 1.31 | 0.0001 | nitrate reductase, subunit alpha, narG |
| NC_008687.1: c1395072-1395761 | 2.08 | 0.0001 | nitrate reductase molybdenum cofactor, narJ |
| NC_008688.1: 207310-209805 | 2.59 | 0.0001 | nitrate reductase catalytic subunit, napA |
| NC_008688.1: 209778-210263 | 2.31 | 0.0001 | nitrate reductase cytochrome c-type subunit, napB |
| NC_008688.1: 210265-210978 | 2.82 | 0.0001 | periplasmic nitrate reductase c-type cytochrome, napC |
| NC_008686.1: 2503999-2505789) | 4.01 | 0.0001 | nitrite reductase |
| NC_008687.1: c1380640-1382598 | 1.63 | 0.0001 | nitrous-oxide reductase |
| NC_008687.1: c1379303-1380634 | 1.52 | 0.0001 | periplasmic copper-binding, nosD |
| NC_008687.1: c1377045-1377620 | 1.31 | 0.0001 | part of nos gene cluster, NosL |
| NC_008687.1: c1377617-1378441 | 1.37 | 0.0001 | nitrous oxide maturation protein, nosY |
| NC_008686.1: 1344725-1345291 | -3.06 | 0.0001 | peroxiredoxin |
| NC_008687.1: c1012304-1012906 | -0.87 | 0.0001 | alkylhydroperoxidase |
| NC_008686.1: 488754-489353 | -2.04 | 0.0001 | superoxide dismutase |
| NC_008686.1: 1723472-1724776 | -1.13 | 0.0001 | Dyp-type peroxidase family protein |


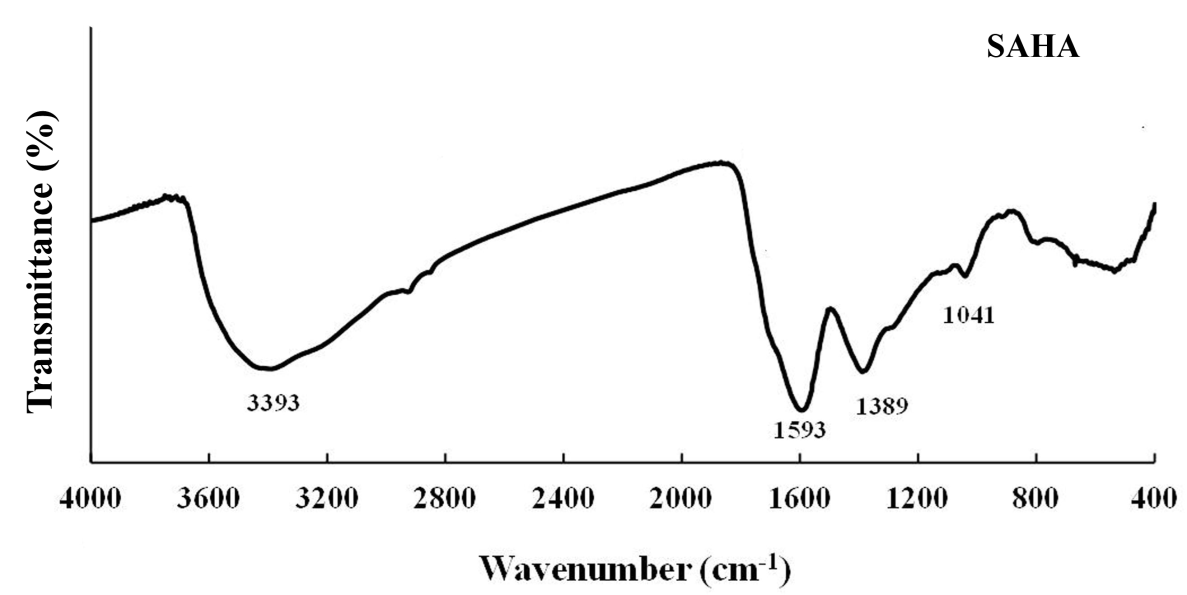


**Fig.S1**. FTIR spectrum of SAHA.


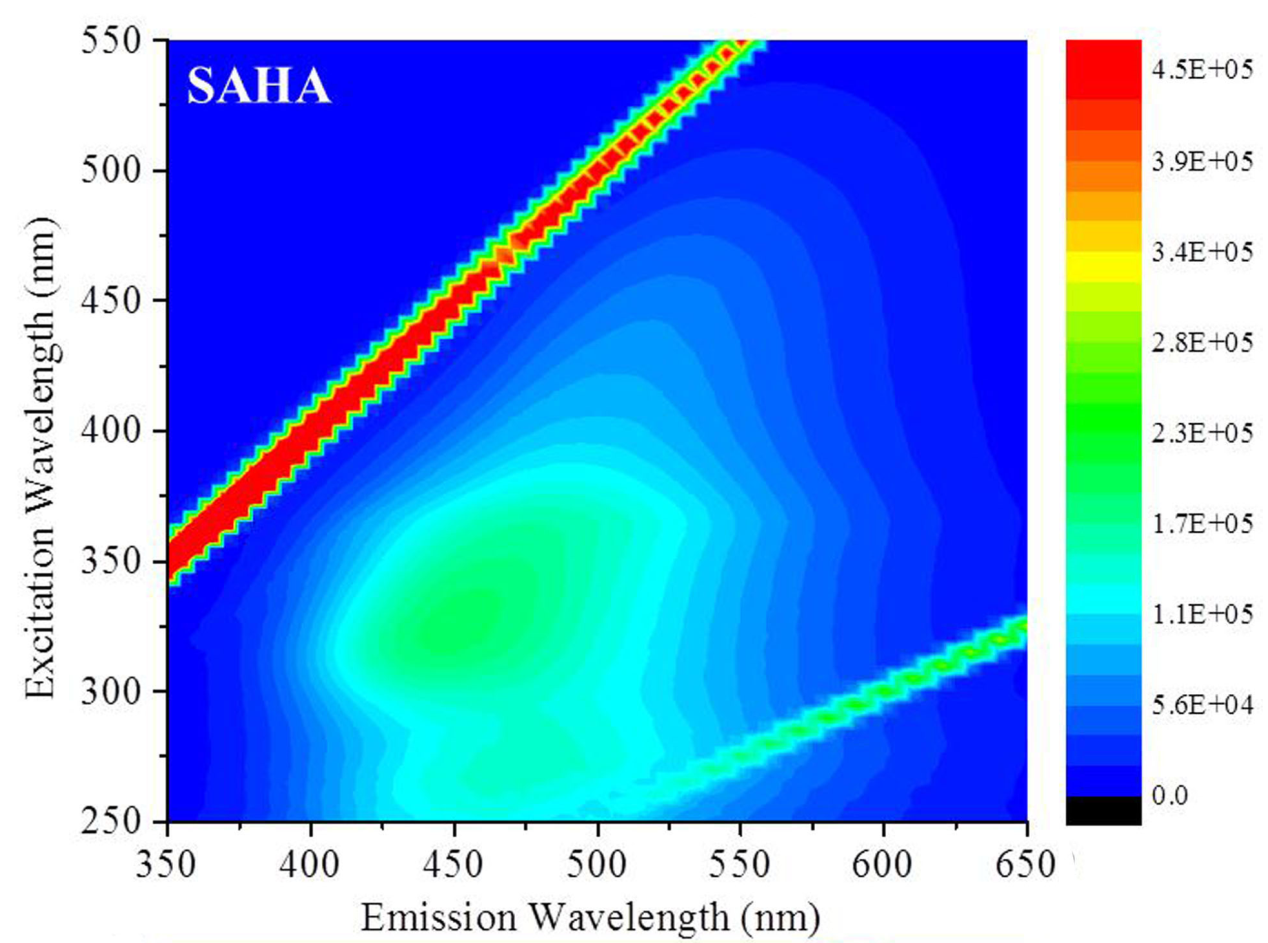


**Fig. S2**. Fluorescence excitation emission matrix spectrum of SAHA. Experiment conditions: pH 7.0 ± 0.1, DOC = 50 mg/L, ionic strength 0.01 M KCl.

FTIR, fluorescence and ^13^C NMR have been widely used to study the spectroscopic characteristics of HA.^11,12^ FTIR analysis is frequently conducted to detect functional groups of humic acid. Excitation emission matrix (EEM) fluorescence is useful to obtain the information on molecular weight and polycondensation of aromatic compounds. ^13^C NMR can quantitatively provide the carbon distribution and chemical environment among the chemical groups.

As exhibited in Fig. S1, four main bands at 3393, 1593, 1389 and 1041 cm^−1^ were detected in SAHA. 3393 cm^−1^ could be assigned to H-bonded OH.^13^ 1593 cm^−1^ could be attributed to aromatic/olefinic C=C, and C=O in carboxyl, ketone and quinone groups.^13,14^ 1389 cm^−1^ CH_2_ could be ascribed to asymmetric bending and carboxylate symmetric stretching motions, C–H deformation of aliphatic and CH_3_ groups).^15^ 1041 cm^−1^ was reported to relate C–O stretching of carbohydrates and alcohols, as well as to C–C stretching motions of aliphatic groups.^16,17^

Fig. S2 shows the excitation-emission matrix (EEM) spectrum of SAHA. One apparent fluorophore (Ex = 315–375 nm and Em = 425–500 nm) was observed in SAHA. The fluorophore mainly composed of carboxylic-like and phenolic-like chromophores,^18,19^ which is consistent with FTIR analysis. On the other hand, the fluorescence peaks at longer wavelengths (such as the one peak) suggest the existence of extended, linearly condensed aromatic ring networks and other unsaturated bonds, which are mainly connected with high molecular weight and humification degree. ^13^C-NMR also indicated that SAHA contained large proportion of aliphatic carbon.


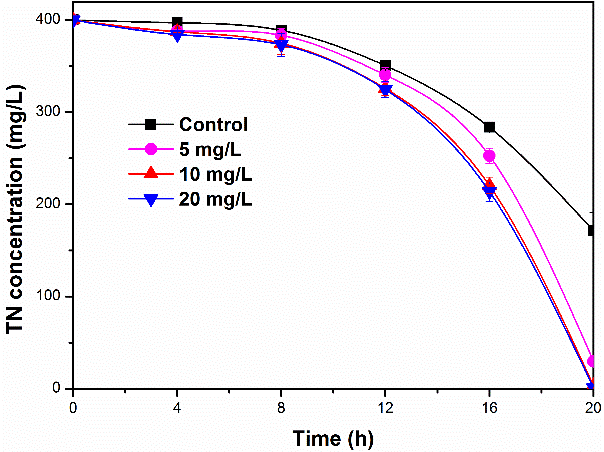

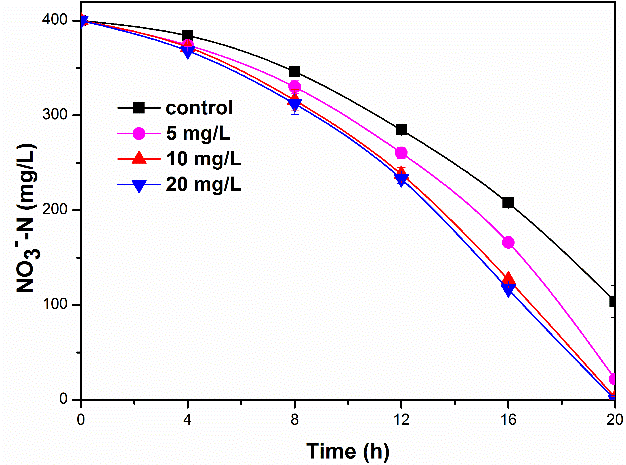


(a)

(b)


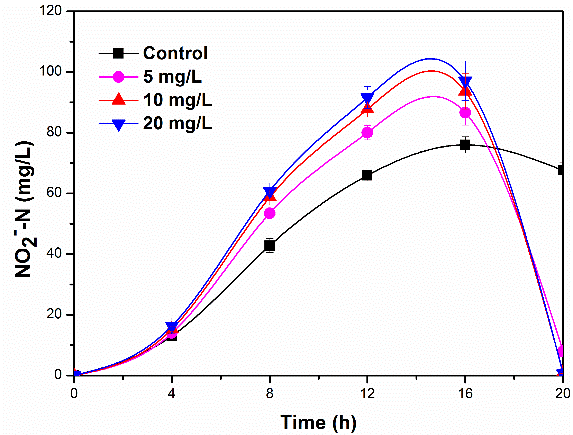

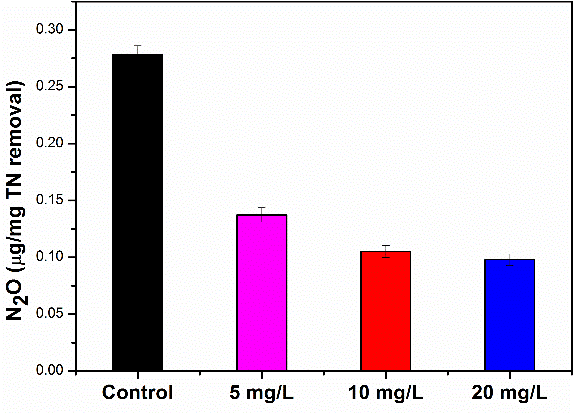


(c)

(d)

**Fig. S3.** The short-term effects of SAHA on the microbial denitrification performance for synthetic wastewater. TN (a), NO_3_^−^-N (b) and NO_2_^−^-N (c), and N_2_O-N release (d). Error bars represent standard deviations of triplicate tests.

In preliminary research, we indeed used 0, 5, 10 and 20 mg/L of SAHA to study the short-term effect of SAHA on the microbial denitrification of synthetic wastewater (Fig. S3). The denitrification performance was examined with the absence and presence of SAHA by running an additional cycle after short-term operations. After 20 h running, the final TN, NO_3_^−^-N, and NO_2_^−^-N and N_2_O concentrations remarkably decreased from 171.4 to 1.0 mg/L, 103.8 to 0.4 mg/L, 67.6 to 0.6 mg/L and 0.28 to 0.10 mg/mg TN removal, respectively, when SAHA concentration increased from 0 to 20 mg/L. For the short-term effects of 10 and 20 mg/L of SAHA on denitrification performance are almost the equally significant, 10 mg/L of SAHA was selected as the target concentration to study the long-term effects on denitrification performance.


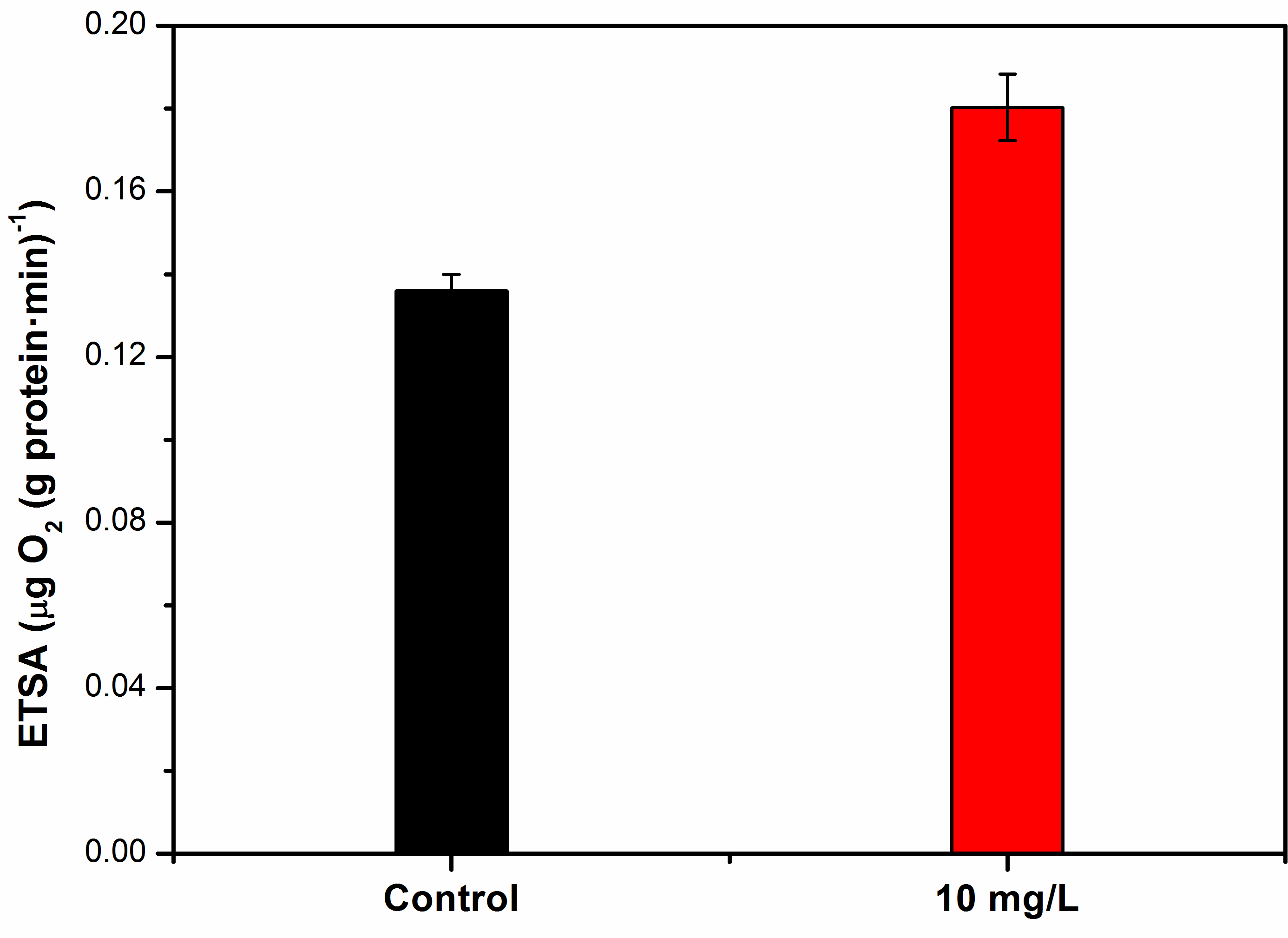


**Fig. S4.** The effects of SAHA on electron transfer system activity (ETSA). Error bars represent standard deviations of triplicate measurements.


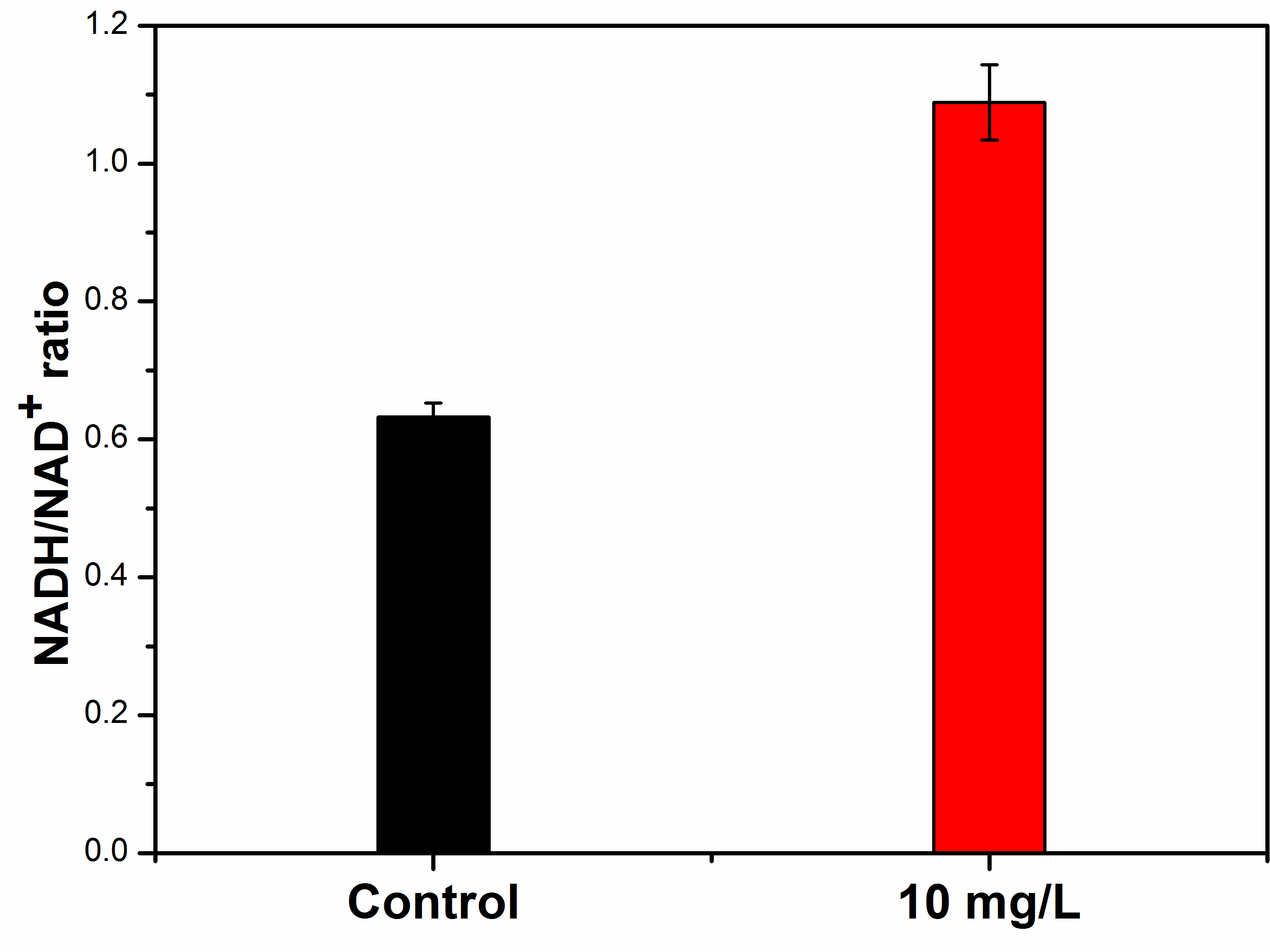


**Fig. S5.** NADH generation in activated sludge after long-term exposure to SAHA. Error bars represent standard deviations of triplicate measurements.

# References

1. Kristjansson, J. & Hollocher, T. First practical assay for soluble nitrous oxide reductase of denitrifying bacteria and a partial kinetic characterization. *Journal of Biological Chemistry.* **255**, 704‒707 (1980).

2. Barak, R., Prasad, K., Shainskaya, A., Wolfe, A. J. & Eisenbach, M. Acetylation of the chemotaxis response regulator CheY by acetyl-CoA synthetase purified from Escherichia coli. *Journal of Molecular Biology*. **342**, 383‒401 (2004).

3. Alp, P. R.. Newsholme, E. A. & Zammit, V. A. Activities of citrate synthase and NAD+-linked and NADP+-linked isocitrate dehydrogenase in muscle from vertebrates and invertebrates. *Biochemical Journal*. **154**, 689‒700 (1976).

4. Müller, M., Hogg, J. F. & De Duve, C. Distribution of tricarboxylic acid cycle enzymes and glyoxylate cycle enzymes between mitochondria and peroxisomes in Tetrahymena pyriformis. *Journal of Biological Chemistry.* **243**, 5385‒5395 (1968).

5. Humphries, K. M. & Szweda, L. I. Selective inactivation of α-ketoglutarate dehydrogenase and pyruvate dehydrogenase: reaction of lipoic acid with 4-hydroxy-2-nonenal. *Biochemistry*. **37**, 15835‒15841 (1998).

6. Lambeth, D. O., Tews, K. N., Adkins, S., Frohlich, D. & Milavetz, B. I. Expression of two succinyl-CoA synthetases with different nucleotide specificities in mammalian tissues. *Journal of Biological Chemistry*. **279**, 36621‒36624 (2004).

7. King, T. E. Preparation of succinate dehydrogenase and reconstitution of succinate oxidase. *Methods in enzymology*. **10**, 322‒331 (1967).

8. Serrano, J. A., Camacho, M. & Bonete, M. J. Operation of glyoxylate cycle in halophilic archaea: presence of malate synthase and isocitrate lyase in Haloferax volcanii. *FEBS letters*. **434**, 13‒16 (1998).

9. San, K.-Y., Bennett, G. N., Berrı́os-Rivera, S. J., Vadali, R. V., Yang, Y.-T., Horton, E., Rudolph, F. B., Sariyar, B. & Blackwood, K. Metabolic engineering through cofactor manipulation and its effects on metabolic flux redistribution in Escherichia coli. *Metabolic Engineering*. **4**, 182‒192 (2004).

10. Broberg, A. A modified method for studies of electron transport system activity in freshwater sediments. *Hydrobiologia*. **120**, 181‒187 (1985).

11. Kang, K. H., Shin, H. S., Park, H. Characterization of humic substances present in landfill leachates with different landfill ages and its implications. *Water Research*. **36**, 4023‒4032 (2002).

12. Amir, S. *et al*. Structural study of humic acids during composting of activated sludge-green waste: Elemental analysis, FTIR and ^13^C NMR. *Journal of Hazardous Materials*. **177**, 524‒529 (2010).

13. Liu K., Chen Y., Xiao N., Zheng X. & Li M. Effect of Humic Acids with Different Characteristics on Fermentative Short-Chain Fatty Acids Production from Waste Activated Sludge. *Environmental Science Technolology.* **49**, 4929–4936 (2015).

14. Amir S. *et al*. Structural study of humic acids during composting of activated sludge-green waste: elemental analysis, FTIR and ^13^C NMR. *Journal of Hazardous Materials*. **177**, 524–529 (2010).

15. Tatzber M. *et al*. FTIR-spectroscopic characterization of humic acids and humin fractions obtained by advanced NaOH, Na_4_P_2_O_7_, and Na_2_CO_3_ extraction procedures. *Journal of Plant Nutrition and Soil Science*. **170**, 522–529 (2007).

16. Chen J., Gu B., LeBoeuf E. J., Pan H. & Dai S. Spectroscopic characterization of the structural and functional properties of natural organic matter fractions. *Chemosphere*. **48**, 59–68 (2002).

17. Li X., Xing M., Yang J. & Huang Z. Compositional and functional features of humic acid-like fractions from vermicomposting of sewage sludge and cow dung. *Journal of hazardous materials*. **185**, 740–748 (2011).

18. McKnight D. M. *et al*. Spectrofluorometric characterization of dissolved organic matter for indication of precursor organic material and aromaticity. *Limnology and Oceanography*. **46**, 38–48 (2001).

19. Yan M., Fu Q., Li D., Gao G. & Wang D. Study of the pH influence on the optical properties of dissolved organic matter using fluorescence excitation–emission matrix and parallel factor analysis. *Journal of Luminescence*. **142,** 103–109 (2013).
